# Use of LMA as ventilatory device for PCT: Our experience

**DOI:** 10.4103/0019-5049.79882

**Published:** 2011

**Authors:** Sneh Lata, Amit Kumar, Adarsh C Swami, Sunny Rupal, Ashwini Sharma

**Affiliations:** Department of Anaesthesiologists’, Fortis Hospital, Mohali, Punjab, India

Sir,

This is to highlight the difficulties experienced and the solutions reached while doing percutaneous tracheostomy (PCT) in our critical care setup. We have done 67 PCTs between March 2008 and Sept 2010. In our neurosurgical ICU, these PCTs were done for patients requiring prolonged ventilation to facilitate better pulmonary toileting and expedite weaning from ventilator. PCT is commonly performed by either Griggs or Ciaglia (multiple/single dilator) techniques.[1] At our institute, we use the Griggs technique. The problems encountered were essentially of airway management during the procedure.

Initially, we used to withdraw the endotracheal tube (ETT) so as to keep the cuff just distal to the glottis, as described in the original technique. While 39 cases were done using ETT cuff distal to the glottis, we faced complications in 6 of the cases. In four of our cases, cuff/ETT wall was punctured by the introducer needle. In one case, there was difficulty in introduction of dilator, while in another one case there was entanglement of guide wire in ETT.

To overcome these problems, we modified the technique and the ETT was withdrawn further, so as to keep the cuff just proximal to the glottis (in 18 cases) and cuff was inflated with 20 ml air and pressed onto glottis to maintain tight seal during IPPV. However, in 3 out of these 18 cases, the ETT got displaced during the crucial steps of the procedure, leading to desaturation which was managed by reintubation.

So, keeping these problems in mind, we used LMA in our last 10 cases without encountering any of the above mentioned complications as seen with ETT with cuff proximal or distal to glottis.[2]

The patients requiring PCT are usually on ventilator for more than 10 days and are having thick / copious secretions. To decrease the risk of aspiration patients are kept NPO for 6 h and, LMA placed after thorough oral suction. The LMA is used for airway management for minimum time (10-15 min, only procedure time). So, aspiration of secretions and inability to maintain PEEP do not pose much of a problem.

We can use fibreoptic bronchoscope also, through LMA,[3] which allows direct step-by-step visualization of procedure and significantly reduces incidence of complications such as posterior tracheal wall tear, false passage, pneumothorax and subcutaneous emphysema thus, making the procedure very safe. In our set up, we could not use fibreoptic endoscope, as it was not readily available. We never encountered any of the above complications in our series. In view of potential advantages of low risk of accidental tube puncture, extubation and no need for an additional assistant, we suggest that LMA, be used, wherever possible as a ventilatory device during PCT.
